# A Case-Control Study on the Association between *Salmonella* Bacteriuria and Cystoscopy

**DOI:** 10.3390/idr13010023

**Published:** 2021-03-01

**Authors:** Eugene Y. H. Yeung

**Affiliations:** Department of Medical Microbiology, The Ottawa Hospital General Campus, The University of Ottawa, Ottawa, ON K1H 8L6, Canada; eugeney@doctors.org.uk

**Keywords:** salmonella, cystoscopy, urinary tract infections, microbial sensitivity tests, bacteremia, *Salmonella enteritidis*

## Abstract

To date, there is only one published report of an outbreak of urinary tract infections by *Salmonella* species after cystoscopy. Disinfection procedures for cystoscope have come into question. The current study aimed to determine the odds of developing *Salmonella* bacteriuria after cystoscopy. A retrospective case-control study was conducted on all patients with *Salmonella* species in urine (case) and blood (control) from 2017 to 2019 in 16 hospitals in Eastern Ontario, Canada. Eight of the 11 patients had cystoscopy prior to *Salmonella* bacteriuria; three of the 74 patients had urological procedures prior to *Salmonella* bacteremia, but none of their procedures were cystoscopy. The odds ratio of urological procedures with *Salmonella* bacteriuria was 63.1 (95% CI 10.9 to 366.6; *p* < 0.0001). In the bacteriuria group, the most frequently identified isolates were *Salmonella enteritidis* (n = 8), followed by *Salmonella oranienburg*, and *Salmonella heidelberg*. Seven of the *S. enteritidis* isolates had identical susceptibilities (ampicillin-sensitive; sulfamethoxazole/trimethoprim-sensitive; ciprofloxacin intermediate). In the bacteremia group, the most frequently identified isolates were *S. enteritidis* (n = 22), followed by *Salmonella typhi*, *S. heidelberg*, *S. oranienburg*, and *Salmonella typhimurium*. The result suggested cystoscopy is a risk factor for *Salmonella* bacteriuria. Identification of *Salmonella* bacteriuria should prompt public health investigations of linkage between cystoscopy and *Salmonella* bacteriuria.

## 1. Introduction

Salmonellosis is a foodborne infectious disease that causes gastroenteritis, bacteremia, and focal metastatic infections. However, *Salmonella* bacteriuria is a very unusual presentation that accounts for <0.1% of all urinary tract infections [1,2]. A study of 19 patients suggested that urological abnormalities are risk factors for *Salmonella* bacteriuria [2]. At one hospital in Spain, four patients underwent cystoscopy and were later identified to have *Salmonella* urinary tract infections between October and November 2014; this unusual presentation suggested the presence of an outbreak [3].

Disinfection procedures for cystoscope have come into question because effective perfusion of disinfectant, rather than immersion alone, is required to reduce microorganism contamination [4]. Outbreaks of cystoscopy infections with *Pseudomonas aeruginosa* [5,6] and *Enterobacter cloacae* [7] were reported in the past. In comparison, there were multiple reports of transmission of *Salmonella* from gastrointestinal endoscopy to hosts [8]. Like other Enterobacteriaceae, *Salmonella* has adhesion factors that facilitate its attachment in hosts and fomites [9]. Like *Pseudomonas* species, *Salmonella* forms a biofilm that facilitates its persistence and resistance in the environment [10]. Furthermore, the Lipid A endotoxin in Gram-negative bacteria make them potential pathogens in human hosts. It is biologically plausible that *Salmonella* species could enter from cystoscopes to human hosts.

In our Eastern Ontario Regional Laboratory Association (EORLA), affiliated with 16 hospitals, we also noted certain patients with *Salmonella* bacteriuria had a history of cystoscopy. Using *Salmonella* bacteremia as a control, the current study aimed to determine the odds of developing *Salmonella* bacteriuria after cystoscopy.

## 2. Materials and Methods

### 2.1. Data Set Creation

The current study followed the guidelines and standards given by the Ottawa Health Science Network Research Ethics Board. The Eastern Ontario Regional Laboratory Association (EORLA) microbiology laboratory used the Cerner Millennium software (Version 2013.04.1.34; Kansas City, MO, USA) to store patients’ laboratory data. This software generated reports that included all patients with *Salmonella* species in urine in a three-year period (1 January 2017 to 31 December 2019). The number of bacteria was quantified using the BD Kiestra™ ReadA Compact imaging acquisition software and established semi-quantitative measurements: <10, 10–100, 100, and >100 × 10^6^ colony-forming unit (CFU)/L [11]. Colony count >100 × 10^6^ CFU/L is generally considered to be significant if patients present with clinical signs and symptoms consistent with urinary tract infection [12]. Patients with *Salmonella* species in their blood in 2017–2019 were used as a control. The microorganisms were identified using matrix-assisted laser desorption ionization-time of flight mass spectrometry (MALDI-TOF MS). Once the MALD-TOF MS identified a microorganism to be *Salmonella* species, the identity of the microorganism was further confirmed with Difco Salmonella O Antiserum Poly A-I and Vi (BD Diagnostics, Sparks, MD, USA), and subsequently, serotyping in Public Health Ontario laboratory. Each patient’s electronic health records (EHRs) were retrospectively reviewed using the software Epic Hyperspace (Version November 2018, Verona, MI, USA). Their prior urological procedures, indications for the procedures, age, gender, microorganisms identified, antibiotic susceptibility results (based on Clinical and Laboratory Standards Institute Kirby–Bauer inhibition zone and E-test minimum inhibitory concentration breakpoints) were recorded in a separate spreadsheet. When the history of urological procedures was not recorded, it was assumed that patients had no prior exposure.

### 2.2. Setting

The EORLA microbiology laboratory, situated at the Ottawa Hospital General campus, Ontario, Canada, is a central laboratory that performed microbiology testing for 16 affiliated hospitals, including the Almonte General Hospital, Arnprior Regional Health, Carleton Place and District Memorial Hospital, Children’s Hospital of Eastern Ontario, Cornwall Community Hospital, Deep River and District Hospital, Glengarry Memorial Hospital in Alexandria, Hawkesbury District General Hospital, Kemptville District Hospital, Montfort Hospital, Pembroke Regional Hospital, Queensway Carleton Hospital, Renfrew Victoria Hospital, St. Francis Memorial Hospital in Barry’s Bay, Ottawa Hospital, and Winchester District Memorial Hospital. Table 1 shows a summary of *Salmonella* bacteriuria and bacteremia patients identified from the laboratory records.

### 2.3. Statistical Analyses

Statistical analyses, including odds ratio (OR), standard error of the log odds ratio (SE), and 95% confidence interval (CI), were performed using online MedCalc software (https://www.medcalc.org/calc/odds_ratio.php, accessed on 20 November 2020). The software generated a standard normal deviate (*z*-value) using the calculation ln(OR)/SE{ln(OR)}; the *p* value represented the area of the normal distribution outside ± *z*. For continuous data, such as age, unpaired, two-tailed Student *t*-test was used (https://www.medcalc.org/calc/comparison_of_means.php, accessed on 20 November 2020). *p* < 0.05 was determined to be statistically significant a priori. A minimum sample size calculation could not be performed because there was no previous data to suggest the OR of *Salmonella* bacteriuria. All patients with *Salmonella* bacteriuria and bacteremia in the study period were included. Only cases in 2017–2019 were included because some of the older records in our laboratory were incomplete.

## 3. Results

Eleven patients were identified to have *Salmonella* bacteriuria, but none of them was identified to have *Salmonella* bacteriuria prior to the study period; eight of these patients had history of cystoscopy prior to the bacteriuria (Table 2). In contrast, 74 patients were identified to have *Salmonella* bacteremia, but none of them was identified to have *Salmonella* bacteremia prior to the study period; three of these patients had history of urological procedures prior to the bacteremia, but none of them had cystoscopy (Table 3). The OR of history of urological procedures with the *Salmonella* bacteriuria group was 63.1 when compared with the *Salmonella* bacteremia group (95% CI 10.9 to 366.6; *z* = 4.62; *p* < 0.0001). The *Salmonella* bacteriuria patients were significantly older than the bacteremia ones (mean age 71 vs. 42 years, respectively; *p* = 0.0005). The bacteriuria patients were predominantly male, significantly different from the bacteremia patients (82% vs. 35%, respectively; OR 8.3; 95% CI 1.7 to 41.3; *z* = 2.59; *p* = 0.0064). None of the *Salmonella* bacteriuria patients had concomitant *Salmonella* species identified in stool culture.

After eliminating the typhoidal *Salmonella* cases (n = 19; *Salmonella typhi* and *Salmonella paratyphi A* and *B*), there were 11 *Salmonella* bacteriuria and 55 *Salmonella* bacteremia patients. The OR of history of urological procedures with the *Salmonella* bacteriuria group was 46.2 when compared with the *Salmonella* bacteremia group (95% CI 7.9 to 270.0; *z* = 4.26; *p* < 0.0001). The *Salmonella* bacteriuria patients remained to be significantly older than the bacteremia patients (mean age 71 vs. 47 years, respectively; *p* = 0.0064). However, the proportion of male patients was no longer significantly different between the two groups (82% vs. 62%, respectively; OR 2.8; 95% CI 0.5 to 14.1; *z* = 1.23; *p* = 0.2178).

In the *Salmonella* bacteriuria group (Table 2), the most frequently identified isolates were *Salmonella enteritidis* (n = 8), followed by *Salmonella oranienburg* (n = 2), and *Salmonella heidelberg* (n = 1). Seven of the eight *S. enteritidis* isolates had identical susceptibilities (ampicillin-sensitive; sulfamethoxazole/trimethoprim-sensitive; ciprofloxacin-intermediate). The one *S. enteritidis* isolate with a different susceptibly profile (ampicillin-sensitive; sulfamethoxazole/trimethoprim-sensitive; ciprofloxacin-sensitive) was from a patient who had cystoscopy exposure after development of *Salmonella* bacteriuria.

In the *Salmonella* bacteremia group (Table 3), the most frequently identified isolates were *S. enteritidis* (n = 22), followed by *S. typhi* (n = 14), *S. heidelberg* (n = 8), *S. oranienburg* (n = 7), and *S. typhimurium* (n = 5). Some of the identified isolates, despite being the same species, have variable susceptibility profiles.

## 4. Discussion

The current study suggested that history of urological procedures was a possible risk factor for *Salmonella* bacteriuria (OR of 63.1 between the *Salmonella* bacteriuria and bacteremia groups). Even after the nontyphoidal *Salmonella* cases were eliminated, the OR remained to be significant at 46.2. It was long believed that *Salmonella* bacteriuria is a rare (<1%), extra-intestinal infectious complication of systemic salmonellosis [13]. The current study suggested that *Salmonella* species could enter the urinary tract through urological manipulation. Although a previous study in Spain reported four patients with *Salmonella* urinary tract infections who had undergone cystoscopy, the study failed to identify the *Salmonella* isolates to species level and compare the isolate susceptibility; three of the four patients also had *Salmonella* species in their stool [3]. It is difficult to determine whether the *Salmonella* isolates from these four patients came from the same source or separately from each of these patients’ fecal contamination.

The current study showed that seven of the eight *Salmonella enteritidis* bacteriuria patients had identical antimicrobial susceptibility profiles. However, the current study failed to prove all these eleven patients’ bacteriuria were from the same source, as different *Salmonella* species were found in four different hospitals.

A study of 19 patients suggested that patients with old age, diabetes mellitus, urologic abnormalities, and immunosuppression were at a higher risk of contracting *Salmonella* bacteriuria [2]. Similarly, the current study showed that patients with *Salmonella* bacteriuria were significantly older than the ones with *Salmonella* bacteremia. Although *Salmonella* species are generally identified more often from females than males [14,15,16,17], *Salmonella* bacteriuria occurred more often in males in the current study.

The major strength of this study was capturing of all incidences of *Salmonella* bacteriuria in 16 affiliated hospitals in Eastern Ontario, Canada, in 2017–2019. This is the largest study to date that investigated the association of *Salmonella* bacteriuria with cystoscopy and had the *Salmonella* species susceptibility data available. The major limitation of the current retrospective study was a lack of thorough interview and examination with each patient to determine their signs and symptoms. However, objective review of EHRs reduced the risk of recall bias and overdiagnosis of urinary tract infections, especially among the elderly [18]. It was assumed that patients had no history of cystoscopy when it was not documented in their EHRs; therefore, the incidence of cystoscopy could be underestimated. Despite the underestimation, the current study showed an OR of 63.1 of *Salmonella* bacteriuria compared with *Salmonella* bacteremia. The difference in age between the *Salmonella* bacteriuria and bacteremia groups could be a confounder but could not be easily controlled in a retrospective study. The microbiology reports did not capture patients with probable contamination in urine (bacteriuria with three or more organisms) and could underestimate the incidence of *Salmonella* bacteriuria. Unfortunately, the current study failed to identify the source of the Salmonella bacteriuria. Based on published literature on infection outbreaks associated with cystoscopy, damages and breaches in reprocessing of cystoscopes were identified as the culprits [5,6,7]. Because of the multi-centered nature of the study, we could not determine whether the disinfection process of cystoscopy in each hospital was consistent with the standard of practice.

Like bacteremia, *Salmonella* isolates in stools could be used as a control. However, nontyphoidal *Salmonella* in stool does not always require antimicrobial treatment [19]; therefore, antimicrobial susceptibility testing of nontyphoidal *Salmonella* isolates in stools were not performed in our laboratory unless requested by clinicians. Using stool isolates as a control would limit the antimicrobial susceptibility data of the isolates identified in the current study. Moreover, cystoscopy would be an unlikely portal of entry in gastrointestinal infection; in contrast, cases of bacteremia had been reported after genitourinary tract manipulation [20,21]. Therefore, *Salmonella* bacteremia was chosen as the control group in the current study.

Salmonellosis is generally a reportable, communicable disease to local public health offices. However, manifestation of *Salmonella* bacteriuria alone may not meet the definition of salmonellosis, since typical presentations are gastroenteritis, bacteremia, and focal metastatic infections. Due to the association of *Salmonella* bacteriuria with cystoscopy in the current study, clinicians and laboratorians should promptly contact local public health offices when they encounter such cases. The current study failed to capture incidences of *Salmonella* bacteriuria identified in the community.

Future quality improvement projects should try to capture community incidences of *Salmonella* bacteriuria. Molecular studies and genome sequencing should also be considered to confirm whether the *Salmonella* isolates are from the same source. For instance, when rare microorganisms are identified from urine samples of patients with history of cystoscopy, laboratories may consider storing the isolates for further testing when needed. Pulsed-field gel electrophoresis (PFGE) is considered the “gold standard” of bacterial typing and widely used for infection control investigations [22], including an outbreak of ertapenem-resistant *Enterobacter cloacae* urinary tract infections due to a contaminated ureteroscope [7]. However, PFGE is a labor-intensive method, and thus, MALDI-TOF MS is being developed as an alternative for bacterial typing [22]. Until a fast, accurate, cheap, and high throughput method is validated for typing, local laboratories may need to send isolates to reference laboratories for further testing.

## 5. Conclusions

The result suggested cystoscopy is a risk factor for *Salmonella* bacteriuria. Identification of *Salmonella* bacteriuria should prompt public health investigations of linkage between cystoscopy and *Salmonella* bacteriuria.

## Figures and Tables

**Table 1:** Demographics of patients identified to have *Salmonella* bacteriuria and bacteremia in 2017–2019.

*Salmonella* Bacteriuria Group	
• Total number of patients	11
• Mean age (year)	71
• Number of males	9
• Number of patients with prior urological procedures	8
• Number of patients with typhoidal *Salmonella* bacteriuria	0
• Number of hospitals identified to have *Salmonella* bacteriuria	4
*Salmonella* bacteremia group	
• Total number of patients	74
• Mean age (year)	42
• Number of males	26
• Number of patients with prior urological procedures	3
• Number of patients with typhoidal *Salmonella* bacteremia	19
• Number of hospitals identified to have *Salmonella* bacteremia	13

**Table 2:** Patients identified to have *Salmonella* bacteriuria in 2017–2019, sorted by names of the microorganisms.

Age (years)	Gender	Source of Urine	Prior Urological Procedures	Procedure Indications	Microorganism	Amount (CFU/L)	Ampicillin Susceptibility	SXT Susceptibility	Ciprofloxacin Susceptibility
52	M	In and out	Cystoscopy	Hematuria	*Salmonella enteritidis*	>100 × 10^6^	S	S	I
52	M	Midstream	Cystoscopy	Hematuria	*S. enteritidis*	>100 × 10^6^	S	S	I
69	M	Midstream	Cystoscopy	Bladder cancer	*S. enteritidis*	>100 × 10^6^	S	S	I
69	M	Cystoscopic	Cystoscopy	Renal cyst; bladder cancer	*S. enteritidis*	<10 × 10^6^	S	S	I
69	M	In and out	Cystoscopy	Renal cyst; bladder cancer	*S. enteritidis*	>100 × 10^6^	S	S	I
79	M	Midstream	Cystoscopy	Bladder cancer	*S. enteritidis*	>100 × 10^6^	S	S	I
86	M	Midstream	None		*S. enteritidis*	>100 × 10^6^	S	S	S
90	M	In and out	None		*S. enteritidis*	>100 × 10^6^	S	S	I
44	F	Midstream	Cystoscopy	Cystocele; stress incontinence	*Salmonella heidelberg*	>100 × 10^6^	S	S	S
83	F	Midstream	Cystoscopy	Urinary incontinence	*Salmonealla oranienburg*	>100 × 10^6^	S	S	S
88	M	Midstream	None		*S. oranienburg*	>100 × 10^6^	S	S	S

Abbreviations: M, male; F, female; CFU, colony-forming unit; SXT, sulfamethoxazole/trimethoprim; S, sensitive; I, intermediate; R, resistant.

**Table 3:** Patients identified to have *Salmonella* bacteremia in 2017–2019, sorted by names of the microorganisms.

Age (years)	Gender	Prior Urological Procedures	Procedure Indications	Microorganism	Ampicillin Susceptibility	SXT Susceptibility	Ciprofloxacin Susceptibility
2	M	None		*Salmonella chester*	S	S	S
23	M	None		*Salmonella choleraesuis* *	R	S	I
20	M	None		*Salmonella eastbourne*	S	S	S
75	M	None		*Salmonella enterica* subsp. *enterica*	R	R	I
85	M	None		*S. enterica* subsp. *enterica*	S	S	I
2	M	None		*S. enterica* subsp. *enterica*	S	S	S
0	M	None		*S. enterica* subsp. *enterica*	S	S	S
57	M	None		*S. enteritidis*	S	S	S
76	F	None		*S. enteritidis*	S	S	S
59	F	None		*S. enteritidis*	S	S	S
33	M	None		*S. enteritidis*	S	S	S
66	F	None		*S. enteritidis*	S	S	S
90	M	None		*S. enteritidis*	S	S	I
63	F	None		*S. enteritidis*	S	S	I
70	M	None		*S. enteritidis*	S	S	S
68	M	None		*S. enteritidis*	S	S	I
86	M	None		*S. enteritidis*	S	S	S
43	M	None		*S. enteritidis*	S	S	S
76	F	Laparoscopic insertion of dialysis catheter	Chronic kidney disease	*S. enteritidis*	S	S	I
11	F	None		*S. enteritidis*	S	S	I
18	M	None		*S. enteritidis*	S	S	S
70	M	None		*S. enteritidis*	S	S	I
58	M	None		*S. enteritidis*	S	S	I
71	M	Dorsal slit	Phimosis	*S. enteritidis*	S	S	S
10	F	None		*S. enteritidis*	S	S	S
75	F	None		*S. enteritidis*	S	S	S
44	M	None		*S. enteritidis*	S	S	I
48	F	None		*S. enteritidis*	S	S	I
86	M	None		*S. enteritidis*	S	S	S
72	M	None		*Salmonella hadar*	S	S	S
22	M	None		*S. heidelberg*	R	S	S
36	M	None		*S. heidelberg*	S	S	S
44	F	None		*S. heidelberg*	S	S	S
19	M	None		*S. heidelberg*	S	S	S
13	F	None		*S. heidelberg*	S	S	S
73	F	None		*S. heidelberg*	S	S	S
1	F	None		*S. heidelberg*	S	S	S
7	M	None		*S. heidelberg*	S	S	S
58	M	None		*Salmonella manhatan*	S	S	S
34	M	None		*Salmonella newport*	S	S	S
72	M	None		*S. oranienburg*	S	S	S
37	F	None		*S. oranienburg*	S	S	S
11	M	None		*S. oranienburg*	S	S	S
48	F	None		*S. oranienburg*	S	S	S
78	F	None		*S. oranienburg*	S	S	I
20	M	None		*S. oranienburg*	S	S	S
33	F	None		*S. oranienburg*	S	S	S
22	F	None		*Salmonella paratyphi A*	S	S	R
33	F	None		*S. paratyphi A*	S	S	R
28	F	None		*S. paratyphi A*	S	S	I
2	M	None		*S. paratyphi B*	S	S	S
28	M	None		*S. paratyphi B*	S	S	S
53	M	None		*Salmonella saintpaul*	S	S	S
33	F	None		*Salmonella stanley*	S	S	S
38	M	None		*Salmonella typhi*	S	S	I
28	M	None		*S. typhi*	S	S	R
24	M	None		*S. typhi*	S	S	I
45	M	None		*S. typhi*	S	S	I
9	M	None		*S. typhi*	S	S	I
19	M	None		*S. typhi*	S	S	I
29	M	None		*S. typhi*	S	S	I
16	F	None		*S. typhi*	R	R	I
25	M	None		*S. typhi*	S	S	S
21	M	None		*S. typhi*	S	S	I
6	M	None		*S. typhi*	S	S	I
42	M	None		*S. typhi*	S	S	R
12	F	None		*S. typhi*	R	R	R
60	M	None		*S. typhi*	S	S	S
69	M	None		*Salmonella typhimurium*	S	S	S
42	M	Renal transplant	End stage renal disease	*S. typhimurium*	S	S	S
73	F	None		*S. typhimurium*	S	S	S
62	M	None		*S. typhimurium*	S	S	S
80	F	None		*S. typhimurium*	R	S	S
22	F	None		*Salmonella virchow*	S	S	S

Abbreviations: M, male; F, female; SXT, sulfamethoxazole/trimethoprim; S, sensitive; I, intermediate; R, resistant. * *Salmonella choleraesuis* was later renamed to *Salmonella enterica.*

## Data Availability

The raw data presented in this study are available on request from the corresponding author. The data are not publicly available due to patient confidentiality.
